# Molecular Characterization of Chronic Lymphocytic Leukemia Patients with a High Number of Losses in 13q14

**DOI:** 10.1371/journal.pone.0048485

**Published:** 2012-11-13

**Authors:** Ana Eugenia Rodríguez, Jose Ángel Hernández, Rocío Benito, Norma C. Gutiérrez, Juan Luis García, María Hernández-Sánchez, Alberto Risueño, M. Eugenia Sarasquete, Encarna Fermiñán, Rosa Fisac, Alfonso García de Coca, Guillermo Martín-Núñez, Natalia de las Heras, Isabel Recio, Oliver Gutiérrez, Javier De Las Rivas, Marcos González, Jesús M. Hernández-Rivas

**Affiliations:** 1 IBSAL,IBMCC, Centro de Investigación del Cáncer, Universidad de Salamanca-CSIC, Salamanca, Spain; 2 Servicio de Hematología, Hospital Universitario Infanta Leonor, Madrid, Spain; 3 Servicio de Hematología, Hospital Clínico Universitario de Salamanca, Salamanca, Spain; 4 Instituto de Estudios de Ciencias de la Salud de Castilla y León, (IECSCYL)–HUSAL, Castilla y León, Spain; 5 Grupo de Bioinformática y Genómica Funcional, IBMCC, Centro de Investigación del Cáncer, Universidad de Salamanca-CSIC, Salamanca, Spain; 6 Celgene Institute for Translational Research Europe (CITRE), Sevilla, Spain; 7 Unidad de Genómica, IBMCC, Centro de Investigación del Cáncer, Universidad de Salamanca-CSIC, Salamanca, Spain; 8 Servicio de Hematología, Hospital General de Segovia, Segovia, Spain; 9 Servicio de Hematología, Hospital Clínico Universitario, Valladolid, Spain; 10 Servicio de Hematología, Hospital Virgen del Puerto, Plasencia, Spain; 11 Servicio de Hematología, Hospital Virgen Blanca, León, Spain; 12 Servicio de Hematología, Hospital Nuestra Señora de Sonsoles, Ávila, Spain; 13 Servicio de Hematología, Hospital del Río Hortega, Valladolid, Spain; University of Navarra, Spain

## Abstract

**Background:**

Patients with chronic lymphocytic leukemia and 13q deletion as their only FISH abnormality could have a different outcome depending on the number of cells displaying this aberration. Thus, cases with a high number of 13q- cells (13q-H) had both shorter overall survival and time to first therapy. The goal of the study was to analyze the genetic profile of 13q-H patients.

**Design and Methods:**

A total of 102 samples were studied, 32 of which served as a validation cohort and five were healthy donors.

**Results:**

Chronic lymphocytic leukemia patients with higher percentages of 13q- cells (>80%) showed a different level of gene expression as compared to patients with lower percentages (<80%, 13q-L). This deregulation affected genes involved in apoptosis and proliferation (BCR and NFkB signaling), leading to increased proliferation and decreased apoptosis in 13q-H patients. Deregulation of several microRNAs, such as miR-15a, miR-155, miR-29a and miR-223, was also observed in these patients. In addition, our study also suggests that the gene expression pattern of 13q-H cases could be similar to the patients with 11q- or 17p-.

**Conclusions:**

This study provides new evidence regarding the heterogeneity of 13q deletion in chronic lymphocytic leukemia patients, showing that apoptosis, proliferation as well as miRNA regulation are involved in cases with higher percentages of 13q- cells.

## Introduction

Chronic lymphocytic leukemia (CLL) is characterized by the progressive accumulation of mature, monoclonal B lymphocytes in the blood, bone marrow (BM) and secondary lymphoid tissues [1]. The clinical course ranges from an indolent disorder with a normal lifespan to a rapidly progressive disease leading to death [2], [3]. The variable clinical course of CLL is driven, at least in part, by the immunogenetic and molecular heterogeneity of the disease [4], [5]. The genomic aberrations and the immunoglobulin (Ig) VH mutation status provide us with two separate genetic parameters of prognostic relevance. Thus, patients whose leukemic cells express unmutated *IgVH* regions (Ig-unmutated CLL) often have progressive disease, whereas those whose leukemic cells express mutated *IgVH* regions (Ig-mutated CLL) more often have an indolent disease [4], [6]. Fluorescent *in situ* hybridization (FISH) can detect genomic abnormalities in more than 80% of CLL cases and the genetic subtypes of CLL show different biological and clinical features [5]. Although unfavorable aberrations (losses on 17p and 11q) are more frequent in the Ig-unmutated subgroup [7]–[9], and favorable aberrations (loss on 13q as a single abnormality) are more frequent in the Ig-mutated subgroup, they have independent value in predicting outcome in CLL [8], [9].

Deletion at 13q14 (13q-) is the most common genomic aberration in CLL. It is present in more than 50% of cases, and is the sole documented cytogenetic abnormality in 36% of the patients. These latter cases are known to have a more favorable clinical course [5], [10]. However, recent data from our group and others, suggest that patients with CLL and 13q deletion as the only FISH abnormality could have a different outcome depending on the number of cells displaying this aberration [11]–[13]. Moreover, previous studies had demonstrated that the percentage of cells displaying a particular cytogenetic abnormality (e.g. loss of *P53)* [14] or antigenic markers (e.g. CD38 or ZAP-70) [7] can be related to prognosis. We have demonstrated that cases with a high number of 13q- cells (13q-H) usually had both shorter overall survival and time to first therapy. However, to the best of our knowledge the molecular characteristics of 13-H CLLs have not been so far analyzed in detail in order to better understand why these patients have a poor outcome.

The value of gene expression profiling (GEP) in the study of CLL is widely accepted. Such studies have identified new prognosis markers such as ZAP-70, LPL, PEG10 and CLLU1. Some of these are already well-established factors used in clinical practice, while the application of others is under study.

As a next step toward elucidation of biological differences within 13q- subgroup, the current study used the Affymetrix Human Exon arrays 1.0 ST, which offer a more fine-grained view of gene expression than the former generation of chips. Thus, the data obtained provide great insights into the biological mechanisms underlying the clinical differences observed in this CLL subgroup [11]–[13].

## Materials and Methods

### Patients

A total of 102 samples were selected for the study, 32 of which served as a validation cohort and five were healthy donors. CLL diagnosis was performed according to the World Health Organization (WHO) classification [15] and the Working Group of National Cancer Institute (NCI) criteria [16]. A complete immunophenotypic analysis by flow cytometry [17] and FISH studies were carried out in all cases. The median age at the time of study was 68 years (range, 35 to 90 years). Most patients were male (66%) and were in Binet clinical stage A (69%), while 26% were in stage B, and the remaining 5% were in stage C. The clinical and biological features of the CLL patients included in the study are shown in Table S1. The study was approved by the local ethical committees “Comité Ético de Investigación Clínica, Hospital Universitario de Salamanca”. Written informed consent was obtained from each patient before they entered the study.

### Methods

#### B cell isolation

Peripheral blood mononuclear cells (PBMCs) were isolated from fresh peripheral blood samples using Ficoll gradient, snap-frozen and stored at –80°C.

For the validation cohort, CD19-positive B cells were purified by magnetically activated cell sorting (MACS) CD19 MicroBeads (Miltenyi Biotec, Bergisch Gladbach, Germany) resulting in a >98% purity, as analyzed by flow cytometry. CD19-positive normal B cells from peripheral blood of five healthy donors served as controls.

#### Genomic aberrations

For the purpose of the study, only samples with one cytogenetic abnormality were included. For the gene expression profile analysis, according to our previous results [11], two groups of patients with 13q- were compared: those in whom 80% or more of cells showed 13q- (13q-H) and those in whom fewer than 80% of cells showed 13q losses (13q-L). The distribution of cases in the study cohort was: 13q-H (n = 25; 36%), 13q-L (n = 27; 39%), normal FISH (nCLL, n = 8; 11%) and 17p−/11q- (n = 10; 14%).

In the validation cohort, the distribution of samples was similar: 13q-H (n = 7; 22%), 13q-L (n = 11; 34%) and nCLL (n = 9; 28%). The remaining five cases were healthy donors.

#### Mutation status of IGVH genes

IGVH genes were amplified and sequenced according to the ERIC recommendations on IGHV gene mutational status analysis in CLL [18].

#### Global gene expression using high density microarrays

Genome-wide expression analysis of the isolated samples was performed using Human Exon 1.0 ST microarrays (Affymetrix). RNA isolation, labeling and microarray hybridization were carried out following the manufacturer’s protocols for the GeneChip platform by *Affymetrix*. Methods included synthesis of first- and second-strand cDNAs, the purification of double-stranded cDNA, synthesis of cRNA by in vitro transcription, recovery and quantization of biotin-labeled cRNA, fragmentation of this cRNA and subsequent hybridization to the microarray slide, post-hybridization washings, and detection of the hybridized cRNAs using a streptavidin-coupled fluorescent dye. Hybridized *Affymetrix* arrays were scanned with an *Affymetrix* Gene-Chip 3000 scanner. Images were generated and features extracted using Affymetrix GCOS Software.

#### Bioinformatic analysis: normalization, signal calculation, significant differential expression, and sample/gene profile clustering

The Robust Microarray Analysis (RMA) algorithm was used for background correction, intra- and inter-microarray normalization, and expression signal calculation [19]. The absolute expression signal for each gene was calculated for each microarray. For the expression signal calculation of the Human Exon arrays we used a new CDF package, called GeneMapper (from GATExplorer) [20], instead of the *Affymetrix* original probe-set definition. This mapping represents an improvement thanks to the reannotation of updated Ensembl gene loci and removal of cross-hybridization noise [20]. It also allows operations to be carried out from the outset using gene identifications (Ensembl IDs) instead of probe-sets (*Affymetrix* IDs). Mapping to genome version Ensembl v53 (assembly NCBI36) was done for these analyses.

Significance Analysis of Microarray (SAM) [21] was used to calculate significant differential expression and to identify the gene probe sets that characterize the samples of each compared state. In this method, permutations provide robust statistical inference of the most significant genes and, by using a false discovery rate (FDR) [22], adjust the raw p-values to take multiple testing into account. An FDR cut-off of <0.05 was used for all the differential expression calculations.

Finally, the Global Test [23] algorithm was used to test the resulting lists of candidate genes associated with 13q-H subgroup. The Global Test allows us to identify the genes that have the global expression pattern most significantly related to the clinical feature studied.

All the bioinformatic analyses were performed with the statistical program R, using the custom packages Bioconductor [24] and GATExplorer [20].

#### Principal component analysis

To explore and represent the differences among the different categories studied (13q-HCLLs, 13q-L CLLs, nCLLs and healthy controls), we applied Principal Component Analysis (PCA) to the expression data sets, using the normalized gene expression matrices of all samples of the validation cohort as the input. The expression matrices were filtered beforehand removing 25% of the least variable genes to avoid noise produced by non-expressed genes (i.e. the remaining 28 806 genes). For each of these genes, the median expression value across samples within each category was calculated. Next, the following formula was designed to calculate the expression values per gene and sample considering their variability within each category:
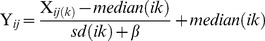
(1)


where Y_ij_ is the PCA input matrix, X_ij_ is the original expression matrix, i is the gene, j the sample, k the category and β = 2 is a small positive constant added to the denominator to ensure that the variance of Y_ij_ is independent of the genes [21]. This formula represents a way of calculating the dispersion of the biological replicates plus its median in each category. In this way, the clustering derived from the principal components includes a small amount of variation between individual samples, highlighting the differences between the categories.

#### Functional analysis and gene annotation

The functional assignment of the genes included in the expression signature of CLL cytogenetic subgroups was carried out by the Database for Annotation, Visualization and Integrated Discovery (DAVID) and the GeneCodis program [25], which identifies concurrent annotations in GO and KEGG, and thereby constructs several groups of genes of functional significance. The most significant biological mechanisms, pathways and functional categories in the data sets of genes selected by statistical analysis were identified through the use of Ingenuity Pathways Analysis Sep2011 (Ingenuity Systems, Mountain View, CA, USA).

#### Gene-specific semi-quantitative PCR

Semi-quantitative SYBRgreen PCR was done in triplicate with iQ™ SYBR® Green Supermix kit (BioRad) using the IQ5 Multicolor Real-Time PCR Detection System (Bio-Rad). Expression data for selected genes were validated in a subset of CLL patients (n = 40). Sense and antisense primers were designed based on the probe-sets used by Affymetrix to synthesize the GeneChip Primer sequences (Table S2). The *ABL1* gene was used as the internal control and the quantification of relative expression [reported as arbitrary units (a.u.)] were performed using the comparative Ct method. The data were not normally distributed, so non-parametric tests were used. Expression levels of the selected genes in both groups (13q-H and 13q-L) were analyzed using the Mann-Whitney U test with a two-tailed value of *P*<0.05 for statistical significance. All tests were performed using SPSS v19.0.

#### Quantification of miRNA expression levels

The expression of selected mature miRNAs was assayed using the Taqman MicroRNA Assays (Applied Biosystems) specific to hsa-mir-15a, hsa-mir-29a, hsa-mir-155 and hsa-mir-223 in 24 CLL patients displaying 13q- according to the manufacturer's recommendations. The Taqman MicroRNA Assays for U43 RNA (RNU43, Applied Biosystems) was used to normalize the relative abundance of miRNA using the 2^−ΔCt^ method. All experiments were performed in duplicate. Expression levels [reported as arbitrary units (a.u.)] of the selected miRNAs in both groups (13q-H and 13q-L) were analyzed using the Mann-Whitney U test in SPSS v19.0. Values of *P*<0.05 were considered statistically significant.

#### Integrative analysis of miRNA and gene expression profile

A summary of the miRNA analysis performed in the study is shown in the Figure S1. miRNAs with significantly different expression (FDR<0.05) between 13q-H and 13q-L were further analyzed to identify the networks and pathway targets. For this purpose, IPA’s microRNA Target Filter, which enables prioritization of experimentally validated and predicted mRNA targets from TargetScan, TarBase, miRecords and the Ingenuity Knowledge Base was used. This tool identified the putative targets for the input miRNAs and then developed the networks among the targets and identified the known and most relevant biological functions, pathways and annotations in this enriched set of target genes. By applying the expression pairing tool, the analysis was focused on targets exhibiting altered expression in our analysis, finding miRNAs and their target genes with opposite or same expression.

## Results

### 13q-H CLLs are Characterized by a Specific Genetic Signature and miRNA Expression

A total of 3 450 genes significantly distinguished 13q-H from 13q-L patients. These comprised 1 244 overexpressed genes and 2 206 underexpressed in the 13q-H group, defining the 13q-H signature. The deregulated genes of the 13q-H signature were annotated and analyzed for the presence of overrepresented “Gene Ontology categories” (Table S3). The most significant overrepresented GO biological processes in 13q-H were related to cell cycle (*P*<0.0001), ribosome (*P*<0.0001) and regulation of transcription (*P*<0.0001). Moreover, 13q-H CLLs had higher levels of expression of *LEF1, BCL2, CARD11, HDAC9, NAFTC1, NFATC2, PAX5, FCRL2* and *SOS1*, while we identified several other genes downregulated in 13q-H, such as *GAS7, E2F1, RRM1, KIT, NP* and *EPOR*. Many of these genes have been reported to be deregulated in CLL, as we confirmed in our analyses that showed overexpression of *LEF1, NFATC1, NFATC2* and *PAX5* in B lymphocytes from CLL patients compared with B lymphocytes from healthy controls (data not shown). PCR results confirmed the microarray data in the analyzed genes such as *GAS7, E2F1* and *FCRL2* (Figure S2).

Moreover, 13q-H CLL patients were also characterized by a striking overrepresentation of deregulated miRNAs. A total of 15 miRNAs were deregulated in 13q-H relative to 13q-L patients. Most of them (eleven) were downregulated while four were upregulated in 13q-H CLL (Table 1).

**Table 1:** miRNAs significantly deregulated between 13q- CLL subgroups (patients with 80% or more of cells with 13q deletion and patients with less than 80% 13q cells).

miRNA	Map	q-value	R fold
Down-regulated			
hsa-mir-1-1*	20q13.33	0.0125	0.7027
hsa-mir-7-1	9q21.32	0.0397	0.5453
hsa-mir-15a	13q14.3	0.0329	0.4917
hsa-mir-29a	7q32.3	0.0354	0.5101
hsa-mir-34a*	1p36.23	0.0366	0.6874
hsa-mir-106b*	7q22.1	0.0280	0.5190
hsa-mir-181b	1q31.3	0.0256	0.6775
hsa-mir-204	9q21.11	0.0294	0.5693
hsa-mir-206	6p12.2	0.0476	0.7077
hsa-mir-221*	Xp11.3	0.0133	0.4622
hsa-mir-223*	Xq12	0.0017	0.1016
**Up-regulated**			
hsa-mir-134	14q32.31	0.0095	1.8096
hsa-mir-105-2	Xq28	0.0182	1.4040
hsa-mir-155*	21q21.3	0.0046	3.7013
hsa-mir-205	1q32.2	0.0161	1.3830

Upregulation or downregulation refers to 13q-H relative to13q-L CLL patients.

miRNA: microRNA.

* deregulation shared with 17p/11q CLL patients.

### Signaling Pathways and Functional Ontology Analyses of Genes Differentially Expressed in 13q-H Patients

**Figure 1:**
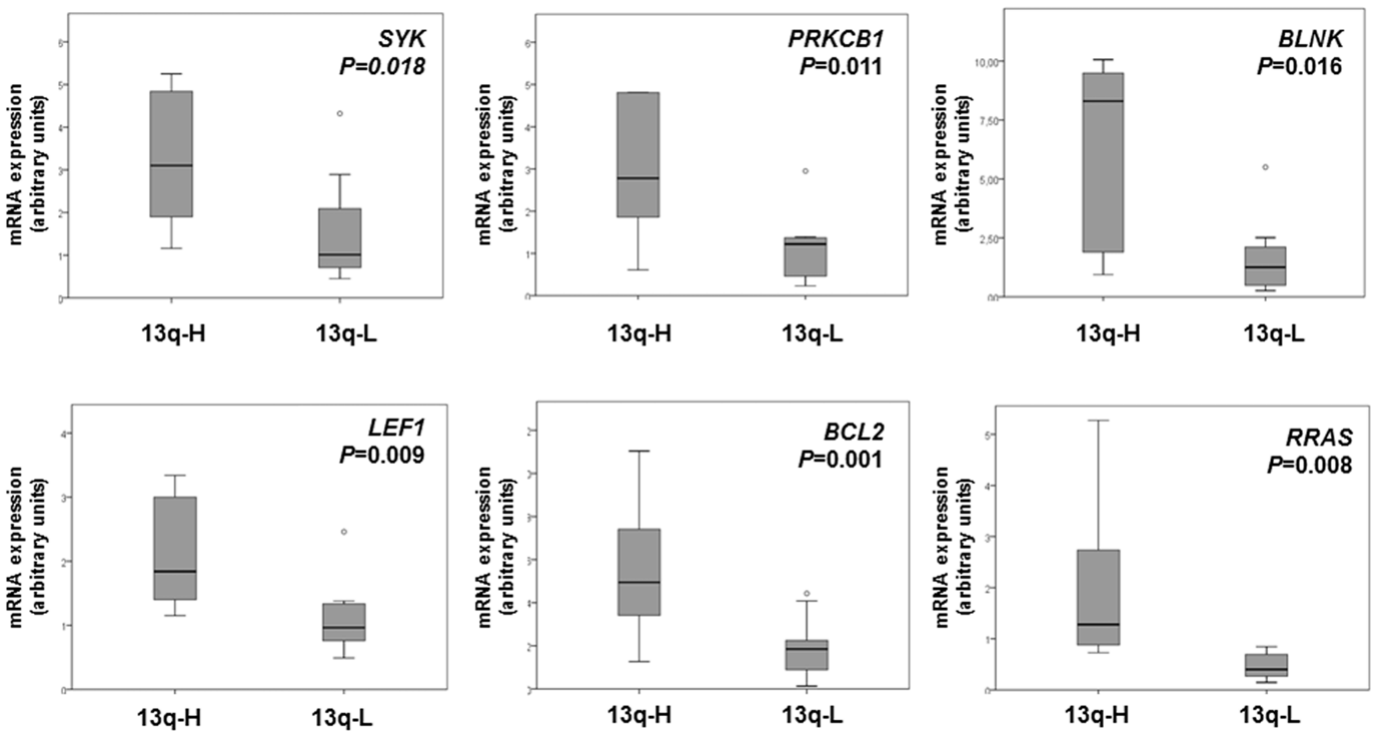
Gene expression levels of genes significantly upregulated in 13q-H CLL patients. Box plot of the expression levels [represented as arbitrary units (a.u.)] of six genes with significantly different expression between 13q-H and 13q-L patients, assessed by semi-quantitative PCR analysis. Box plots show the relative upregulation of BCR (SYK, PRKCB1 and BLNK), proliferation (LEF1 and RRAS2) and antiapoptotic (BCL2) related genes in patients with a high number of 13q- cells compared with CLL patients with lower percentages of losses in 13q. The thick line inside the box plot indicates the median expression levels and the box shows the 25th and 75th percentiles, while the whiskers show the maximum and minimum values. Outliers (extreme values falling out of the main distribution) are represented by open circles. Statistical significance was determined using the Mann-Whitney U test (*P*<0.05).

To determine the biological significance of the deregulated genes, a further analysis of the 3 450 deregulated genes characterizing the 13q-H CLL was carried out, revealing in this group of patients the involvement of several pathways (Table 2). These pathways are primarily related to cell proliferation, apoptosis and cell signaling. Thus the BCR pathway was upregulated in 13q-H CLL patients. In fact, 21 genes from this pathway were overexpressed in 13q-H CLLs, some of which, such as *SYK*, *BLNK* and *PRKCB1*, were previously related to CLL pathogenesis (Figure S3). We also observed an imbalance in proliferation and apoptosis in 13q-H patients, due to upregulation of antiapoptotic genes (*BCL2*) and decreased expression of proapoptotic genes (*RASSF5*, *BAD*, *CASP8*, *CASP10*, *FAS*) in 13q-H patients. Moreover, our analysis showed an overexpression of genes promoting proliferation, such as *LEF1*, *E2F5* and *RRAS2*. To ensure that the gene expression profiles accurately reflected the upregulation of BCR signaling pathway and the deregulation of apoptosis-related genes, representative genes that were differentially expressed in 13q-H patients were assessed by semi-quantitative SYBRgreen PCR analysis. These included *SYK*, *BLNK* and *PRKCB1* (BCR signaling pathway), *BCL2* (apoptosis) and *LEF1* and *RRAS2* (proliferation). The semi-quantitative PCR results were in close agreement with the microarray data (Figure 1) confirming the overexpression of these genes in 13q-H CLLs compared with 13q-L. Western blot analysis should be made for a more concluding validation after mRNA screening. Unfortunately, due to the lack of material, this was not possible in this study.

**Table 2:** Enriched functional analysis of the 3450 genes differentially expressed between the two 13q- patient subgroups: 1244 genes were upregulated (i) and 2206 genes were downregulated (ii) in CLL patients with ≥80% cells displaying 13q deletion.

	Up-regulated				Down-regulated		
i.	Ingenuity Canonical Pathway	p-value	Up-regulated genes	ii.	Ingenuity Canonical Pathway	p-value	Down-regulated genes
	EIF2 Signaling	1,70E-07	RPL24,RPL27A,RPL26,RPS11,RPS27,RPS3A,SOS1,RPL35,RPL19,RPL13,RPL39L,RPL34,RPL27,RPL21,RPS19,RPL23A,RPS29,RPL36,RRAS2,RPS13,RPL26L1,RPL32,RPS25,RPS15A,RPL13A,RPS27A,RPL41,RPS14,RPSA		Mitotic Roles of Polo-Like Kinase	1,35E-05	KIF23,CDC25C,ESPL1,CDC20,PPP2CA,PRC1,CDC7 (includes EG:12545),CCNB2,CDC23,PLK1,PPP2R5A,CDK1,CCNB1,SLK,HSP90B1,PLK4,PKMYT1,PPP2R1B,KIF11,CDC27,CDC25A
	B Cell Receptor Signaling	1,95E-05	MAP2K6,BLNK,MAP3K14,MAP3K9,CD19,CD79B,BAD,POU2F2,IKBKE,NFATC1,FCGR2B,PTEN,MAP3K12,RRAS2,CAMK2D,SYK,SOS1,CD22,NFATC2,PIK3AP1,PPP3CA,PRKCB		Cell Cycle Control of Chromosomal Replication	2,75E-05	MCM6,CDC45,CDT1,CDC6,CDC7 (includes EG:12545),CDK6,ORC6,MCM4,MCM3,MCM2,CDK2,MCM7,ORC1
	PI3K Signaling in B Lymphocytes	6,92E-05	BLNK,CD19,CD79B,IKBKE,NFATC1,FCGR2B,PRKCZ,PTEN,BLK,CAMK2D,RRAS2,CD180,SYK,IRS1,SH2B2,NFATC2,PIK3AP1,PPP3CA,PRKCB		Caveolar-mediated Endocytosis Signaling	6,17E-04	FYN,ITGA2B,ITSN1,RAB5A,ACTB,COPA,ITGA6,ITGA5,COPB1,ACTG1,COPG,COPB2,DYRK3,ITGB2,ITGAE,ITGAM,ITGA9,ITGAV,HLA-C,ITGA4
	CD27 Signaling in Lymphocytes	2,75E-03	MAP2K6,MAP3K12,MAP3K9,MAP3K14,CD70,IKBKE,TRAF5,CD27,MAP2K5		Glycolysis/Gluconeogenesis	7,94E-04	PGK1,ALDH4A1,PGM2,PKLR,GAPDH,PGM1,BPGM,PDHA1,HK1,ALDH2,GPI,HK2,ALDH1A1,DHRS9,ENO1,DLAT,DLD,FBP1,ALDH3B1,LDHA,ACSL1
	mTOR Signaling	5,37E-03	VEGFB,RHOC,RPS19,RPS11,PRKCZ,RPS29,RPS27,RPS3A,RRAS2,RPS13,IRS1,GPLD1,RPS15A,RPS25,GNB1L,RPS27A,RPS14,RPSA,PRKCB		Integrin Signaling	1,12E-04	RAP2B,RAF1,FYN,ITGA2B,ARHGAP26,TSPAN7,PIK3R1,PIK3R5,PPP1CB,NCK1,SHC1,ITGAE,PARVB,ARF6,WASL,RHOG,ITGA9,ARF4,PIK3CG,RHOU,ITGAV,VCL,MAP2K1,ACTN1,ITGA4,PXN,NRAS,ASAP1,CRKL,ACTB,ITGA6,TSPAN2,ITGA5,ACTG1,ITGB2,ARF1,ITGAM,TLN2,ZYX,PIK3CB,ACTN4,CTTN
	Role of JAK1 and JAK3 in γc Cytokine Signaling	6,76E-03	BLNK,IL2RG,RRAS2,IRS1,SYK,SH2B2,JAK2,STAT1,IL7		Cyclins and Cell Cycle Regulation	1,17E-03	RAF1,E2F4,CCNE2,TFDP1,HDAC2,PPP2CA,SUV39H1,CDK6,CDKN2C,CCNB2,E2F3,PPP2R5A,CDK1,CCNB1,CCNA2,CCNE1,E2F1,PPP2R1B,E2F2,CDK2,CDC25A
	Nucleotide Excision Repair Pathway	1,20E-02	ERCC4,ERCC1,GTF2H1,ERCC2,MNAT1,XPA		Role of CHK Proteins in Cell Cycle Checkpoint Control	1,32E-03	CDC25C,E2F4,E2F1,RFC2,E2F3,BRCA1,CDK1,E2F2,CDK2,CDC25A,CHEK1,RFC3
	Regulation of eIF4 and p70S6K Signaling	1,66E-02	RPS19,RPS11,PRKCZ,RPS29,RPS27,RRAS2,RPS3A,RPS13,IRS1,SOS1,RPS25,RPS15A,RPS27A,RPS14,RPSA		Nicotinate and Nicotinamide Metabolism	1,66E-03	DAPK1,PRKCQ,SGK1,MAPK6,CSNK1A1,CDK6,CSNK1D,PLK1,TTK,CDK1,SACM1L,VNN1,NEK2,ARAF,GRK6,PRKAA1,PNP,CD38,HIPK1,MAP2K1,NMNAT3,CDK2,BST1,DUSP16
	Phospholipase C Signaling	1,86E-02	BLNK,PEBP1,CD79B,RHOC,MEF2A,HDAC9,NFATC1,FCGR2B,MYL6B,PRKCZ,RRAS2,SYK,SOS1,GPLD1,NFATC2,MEF2C,GNB1L,ARHGEF9,PPP3CA,PRKCB		Inositol Phosphate Metabolism	1,78E-03	MINPP1,SGK1,PIK3R1,PIK3R5,CSNK1A1,TTK,OCRL,NEK2,PIK3CG,PRKAA1,PLCB1,IMPA2,PI4K2B,HIPK1,MAP2K1,PMPCA,MTMR3,DAPK1,IMPA1,PRKCQ,MTMR14,MAPK6,CDK6,CSNK1D,PLK1,CDK1,ARAF,SYNJ1,GRK6,PIK3CB,CDK2
	PKCθ Signaling in T Lymphocytes	1,86E-02	MAP3K12,MAP3K9,MAP3K14,POU2F1,RRAS2,CAMK2D,SOS1,NFATC2,IKBKE,NFATC1,CARD11,PPP3CA		Cell Cycle Regulation by BTG Family Proteins	4,57E-03	CCNE2,E2F4,CCNE1,PPP2CA,E2F1,E2F3,PPP2R1B,CCRN4L,E2F2,CDK2,PPP2R5A
	April Mediated Signaling	2,34E-02	MAP3K14,NFATC2,IKBKE,NFATC1,TRAF5,TNFRSF17		Clathrin-mediated Endocytosis Signaling	6,61E-03	AP2A1,STON2,PIK3R1,PIK3R5,PDGFC,VEGFA,ARF6,ARRB1,WASL,SNX9,PIK3CG,DAB2,CSNK2B,AAK1,AP2M1,RAB5A,ACTB,CHP,CLTC,RAB7A,ITGA5,ACTG1,TSG101,ITGB2,ARRB2,LDLR,SYNJ1,TFRC,PIK3CB,DNM1L,CTTN
	Interferon Signaling	2,63E-02	OAS1,IFI35,JAK2,STAT1,BCL2		Sphingolipid Metabolism	6,76E-03	LASS6,GLA,GALC,SGMS2,ASAH1,SACM1L,LASS2,VNN1,LPIN1,GBA,SMPD4,GLB1,PPAP2B,SPHK1,ARSB,FUT4,KDSR,DUSP16
	IL-4 Signaling	2,69E-02	IL2RG,RRAS2,IRS1,SOS1,NFATC2,NFATC1,JAK2,FCER2		Role of BRCA1 in DNA Damage Response	7,08E-03	E2F4,BARD1,RBBP8,PLK1,E2F3,CHEK1,RAD51,GADD45A,E2F1,RFC2,BRIP1,BRCA1,HLTF,E2F2,RFC3
	B Cell Activating Factor Signaling	2,95E-02	MAP3K14,NFATC2,IKBKE,NFATC1,TRAF5,TNFRSF17		Protein Ubiquitination Pathway	7,94E-03	USP24,USP14,USP12,UBE2H,PSMD7,CDC20,USP20,DNAJC3,CDC23,HSPA5,USP39,SMURF1,USP3,HSP90B1,USP42,USP47,NEDD4L,BRCA1,PSMC2,HLA-C,DNAJB12,USP15,MED20,USP36,USP38,HSPA9,USP19,PSMD6,PSMD5,HSPD1,PSMD3,USP1,UBE2D1,NEDD4,TRAF6,PSMD11,DNAJC5,USP4,PSMD2,DNAJB11,PSMD12,HSPA13,BAP1,PSMD1,DNAJB6,PSMC3,UBE2C,BIRC2
	NF-κB Signaling	6,46E-02	MAP2K6,MAP3K14,FLT1,BMPR2,PRKCZ,TNFRSF17,TLR10,RRAS2,BMPR1A,TLR6,TLR7,TRAF5,CARD11,PRKCB				

### miRNA Deregulation in 13q-H CLL Patients

**Figure 2:**
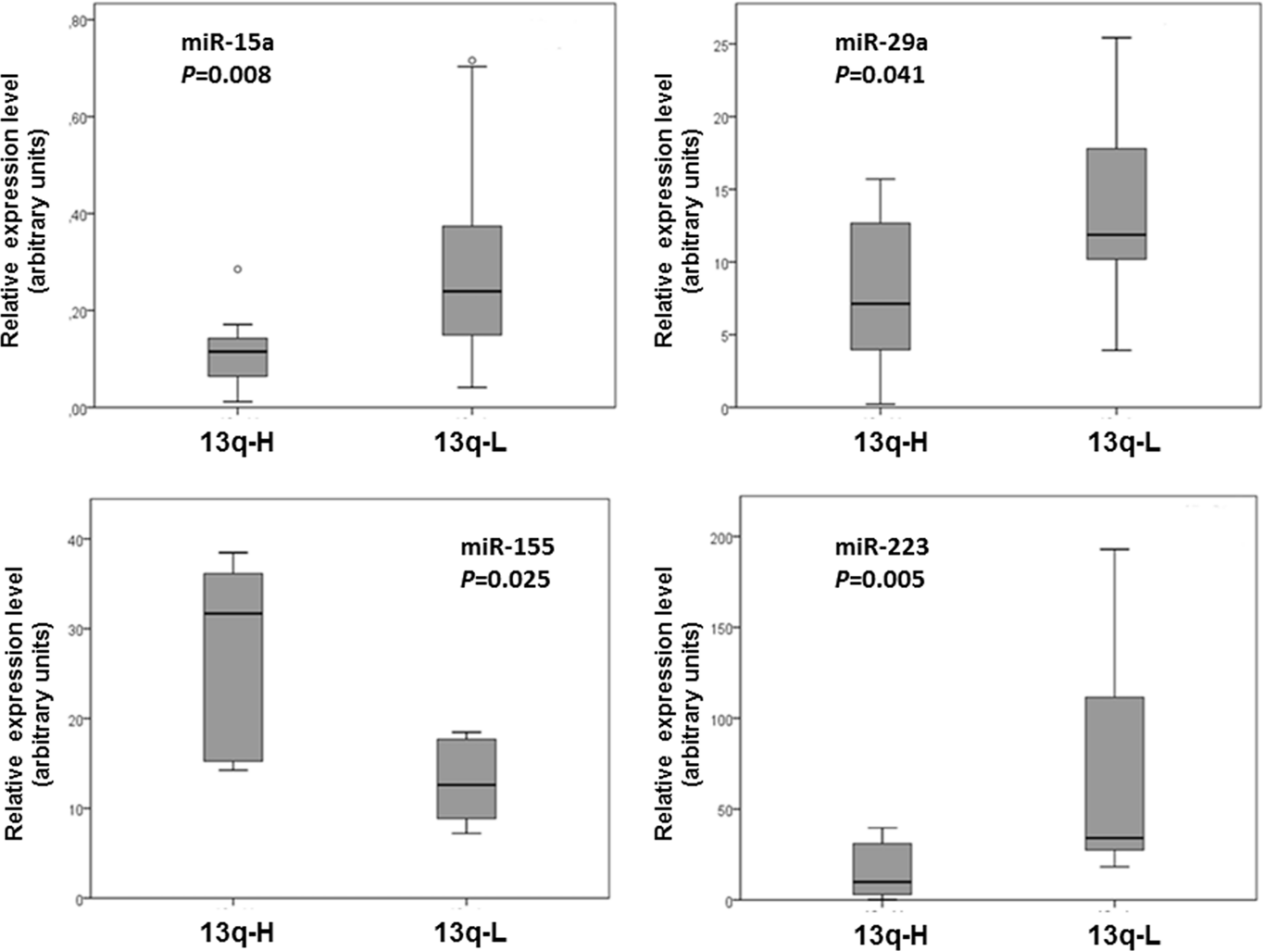
Quantitative RT-PCR validation for miR-15a, miR-29a, miR-155 and miR-223 in independent CLL patients. Relative expression of miR-15a, miR-29a, miR-155 and miR-223 [represented as arbitrary units (a.u.)] was evaluated by individual TaqMan miRNA assays performed in duplicate and normalized to RNU43 (2^−dCt^). Box plots indicate the median value (horizontal line) and the 25^th^–75^th^ percentile range (box) while whiskers showing the maximum and minimum values. Values outside this range are shown as outliers (open circles). *P*-values were determined by the Mann-Whitney U test. In every case, miRNAs downregulated in 13q-H CLL patients relative to 13q-L patients were also found to be downregulated by quantitative RT-PCR. Similar observations were made for miR-155, which was upregulated in 13q-H patients. All comparisons were statistically significant (*P*<0.05).

The analysis of miRNA expression in 13q-H and 13q-L CLL patients revealed that fifteen miRNAs were deregulated in 13q-H CLL patients: hsa-miR-155 was the most highly upregulated miRNA (Rfold = 3.70), while hsa-miR-223 was the most significantly downregulated (Rfold = 0.10). Four of the deregulated miRNAs (miR-15a, miR-29a, miR-155 and miR- 223) were further assayed by quantitative RT-PCR for validation purposes in 24 CLL samples displaying 13q-. Results confirmed the upregulation of miR-155 and the downregulation of miR-15a, miR-29a and miR- 223 in 13q-H samples relative to 13q-L (Figure 2).

**Figure 3:**
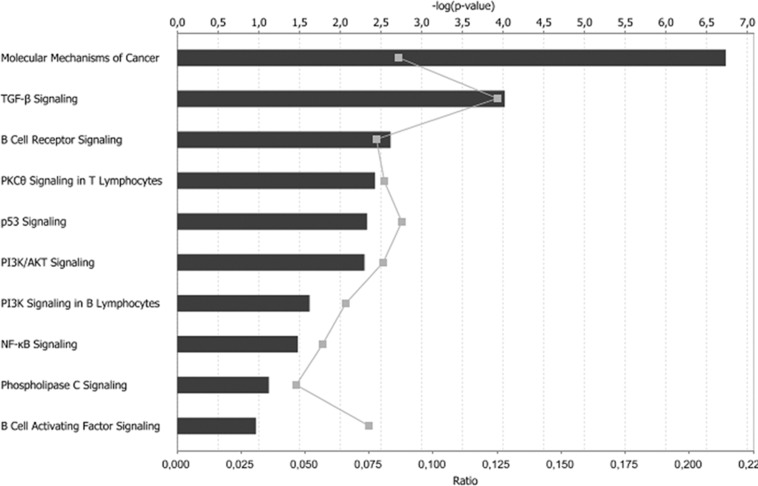
Most significant cellular functions affected by the deregulation of miRNAs in 13q-H CLL patients. 432 out of the 1027 predicted mRNA target genes of the deregulated miRNAs in 13q-H CLL patients appeared also deregulated in our analysis. A functional enrichement analysis was performed in this dataset. Category names are presented on the vertical axis. Of note, B cell receptor signaling and NF-kB signaling were among the most significant cellular functions affected. The significance of the association between the dataset and the canonical pathway was measured in two ways: (1) the ratio of the number of genes from the dataset that met the expression value cut-off that map onto the pathway divided by the total number of molecules that exist in the canonical pathway, represented by grey squares in the graph and (2) the *P*-value determining the probability of the association between the genes in the dataset and the canonical pathway, calculated by Fisher's exact test. The horizontal axis on the top indicates the −log (*P* value) and the horizontal axis at the bottom, the ratio. In both cases, the higher value indicates the higher significance.

The influence of these deregulated miRNAs on 13q- patients was assessed (Figure S2). Specifically, we investigated whether observed changes in miRNAs were correlated with changes in the expression of genes. Therefore the post-transcriptional regulatory network of miRNA and genes in CLL patients with more than 80% of 13q- cells was carried out by analyzing the miRNA-mRNA relationships. A total of 1 027 mRNA putative targets with altered expression in 13q-H CLL patients were found (Table S4). Indeed, because miRNAs tend to downregulate the target genes, we focused our study on the subset of 11 miRNAs selected for analysis in IPA and the 432 genes predicted to be regulated by them and characterized by expression profiles stringly anticorrelated. Functional analysis revealed that transcription was the cell function most strongly affected by these miRNAs, with a total of 97 genes affected by the 11 selected miRNAs. Modification of proteins (n = 41), proliferation of immune cells (n = 34), and activation of protein binding sites (n = 32) were other important functions affected by these miRNAs (Table S5). Finally we performed a functional analysis of the 11 miRNAs and their 432 putative targets. The pathway analysis demonstrated that, again, B cell receptor signaling, PI3K signaling and NFkB signaling were among the most strongly affected pathways in 13q-H patients (Figure 3), highlighting the importance of miRNA regulation in CLL. MiR-155, the most overexpressed miRNA in 13q-H, was negatively correlated with the expression of 90 of the 182 expected genes (49%), demonstrating a relationship between miRNA and gene deregulation. Interestingly, most of these putative targets were assigned to the functional categories of transcription regulation (*P* = 0.002). Moreover, we found several miRNAs whose targets that were experimentally observed or predicted with high confidence were strongly related to CLLs such as *BCL2* (miR-15, miR-206, miR-106b and miR-34a), *TCL1A* (miR-29a) and *LEF1* (miR-34a) (Table 3).

**Table 3:** Most significant target genes affected by deregulation in miRNA in 13q-H CLL patients.

Target			miRNA	
Symbol	Fold Change	B-cells related pathways	ID	Fold Change
			hsa-mir-206	0.708
			hsa-mir-15a	0.492
*BCL2*	2.132	Apoptosis	hsa-mir-106b	0.519
			hsa-mir-204	0.569
			hsa-mir-34a	0.687
			hsa-mir-206	0.708
*E2F5*	2.624	DNA Damage Response	hsa-mir-106b	0.519
			hsa-mir-34a	0.687
*FOS*	0.447	B Cell Activating Factor,CD27	hsa-mir-155	3.701
*LEF1*	2.835	ILK, Wnt	hsa-mir-34a	0.687
*MAP2K6*	3.558	BCR,CD27	hsa-mir-29a	0.510
*MAP3K12*	1.254	BCR,CD27	hsa-mir-106b	0.519
*MAP3K14*	1.348	Apoptosis,B Cell Activating Factor,BCR,CD27	hsa-mir-106b	0.519
*MAP3K9*	1.400	BCR,CD27	hsa-mir-106b	0.519
*MYD88*	0.752	NF-κB,Toll-like Receptor	hsa-mir-155	3.701
*PLCB1*	0.773	PI3K	hsa-mir-205	1.383
*RRAS2*	1.931	Apoptosis, BCR	hsa-mir-223	0.102
			hsa-mir-15a	0.492
*SOS1*	2.352	BCR	hsa-mir-106b	0.519
			hsa-mir-204	0.569
*TCL1A*	7.848	Akt	hsa-mir-29a	0.510

### The GEP of 13q-H CLL Patients is Similar to that in CLL Patients with 11q or 17p Losses

**Figure 4:**
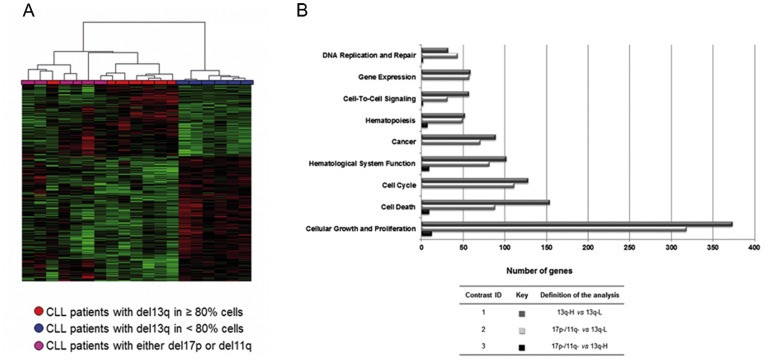
Differential expression analysis followed by pathway analysis revealed commonly deregulated biological processes in CLL patients with a high load of 13q- cells, 17p- and 11q-. A. Heatmap of 3450 differentially expressed genes in CLL patients with a high number of losses in 13q (red), losses in 17p or 11q (magenta) and a low number of losses in 13q (blue). Differentially regulated genes were identified using Significance Analysis of Microarray (SAM), with a false discovery rate 5%, followed by the Global Test algorithm to test the candidate genes associated with the group of patients with a high number of losses. Individual patients are arranged in columns with the expression level for each gene across rows. Normalized gene expression values are color-coded (standard deviation from mean): red and green indicate high and low expression, respectively. All patients with 13q-L were clustered on the right side of the map in a homogeneous manner and separately from 13q-H and 17p−/11q-, which clustered together, showing that the gene expression profile (GEP) of CLL cases with higher percentages of 13q- cells is similar to that of 17p- and 11q-, while CLL patients with lower percentages of 13q- cells had a different gene profile. **B. Commonly deregulated biological functions in 13q-H and 17p−/11q- CLL patients compared with 13q-L CLL subgroup.** Biological function names are presented on the vertical axis and the number of deregulated genes involved in each function, in the horizontal one. Fisher's exact test was used to examine the probability of the association between the genes in the dataset and the functional category. The color-coded bar plot (dark grey, light grey and black bars) depicts the analysis results. 13q-H patients showed marked differences in the expression of genes related to several cellular functions compared with 13q-L CLL patients (comparison 1, dark grey bars). In addition, most of these cellular functions were also deregulated in comparison with high-risk cytogenetic subgroups (17p- and 11q-) and 13q-L CLL patients (comparison 2, light grey bars). Thus, 13q-H, 17p- and 11q- patients share the deregulation of several important functions relative to 13q-L patients. Furthermore, a small number of genes related to cell cycle, cell growth and DNA repair (comparison 3, black bars) were found to be differentially expressed in the 13q-H group in a comparison of this subgroup of patients and high-risk cytogenetic subgroups.

We also analyzed the gene signature of CLL high risk cytogenetic subgroups in comparison with 13q- patients. Surprisingly, a significant number of deregulated genes were found to be shared between the genes that differentiate 13q subgroups and 13q-L and high risk subgroup of patients. That is, the GEP of 13q-H CLL patients resembled the gene expression pattern of patients with 17p- or 11q- abnormalities (Figure 4A). In fact, both subgroups of CLL patients (13q-H and the 17p- and 11q- subgroup) shared 1 325 genes (46%) of the deregulated genes in the global analysis including all CLL subtypes. By contrast, the comparison between the GEP of 13q-H patients and those with losses in either 17p or 11q showed fewer differences in expression (Figure S4).

To evaluate the biological significance of the observed similarity between the 13q-H and the 17p−/11q- signatures, we used the Ingenuity Pathway Analysis comparative tool, which facilitates the functional comparison of several panels of differentially expressed genes. Thus, we identified several commonly deregulated biological functions in both gene signatures (Figure 4B), such as cell cycle, cell death, cellular growth and proliferation. Finally, pathway analysis was performed on those genes commonly upregulated or downregulated in 13q-H, 17p- and 11q- patients in comparison with the 13q-L subgroup (Table S6). In accordance with the comparative analysis results, several commonly deregulated pathways of relevance in CLL pathogenesis were observed. The most significant of these were the B cell receptor signaling pathway for commonly upregulated genes, and the cell cycle control of chromosomal replication pathway for commonly downregulated genes in patients showing 13q-H, 17p- or 11q- (Table S6). The expression of the *TCL1* gene had one of the lowest q-values (0.002) with higher expression levels in patients with 13q-H, 17p- and 11q-. Of note, 13q-H, 17p- and 11q- patients also shared the deregulation of several miRNAs (Table 1).

### Genome-wide Expression Differentiates 13q-H CLLs from 13q-L CLLs and Controls

To validate the differences observed between the subgroups of 13q- CLL patients and get a visualization of these, we applied the Principal Component Analysis (PCA) in an independent series of patients. The clustering algorithm of PCA reduces complex multidimensional data to a few specified dimensions so that it can be visualized effectively. For a better characterization of the differences, we included in this cohort patients with normal FISH (nCLL) and healthy donors as two different types of controls.

**Figure 5:**
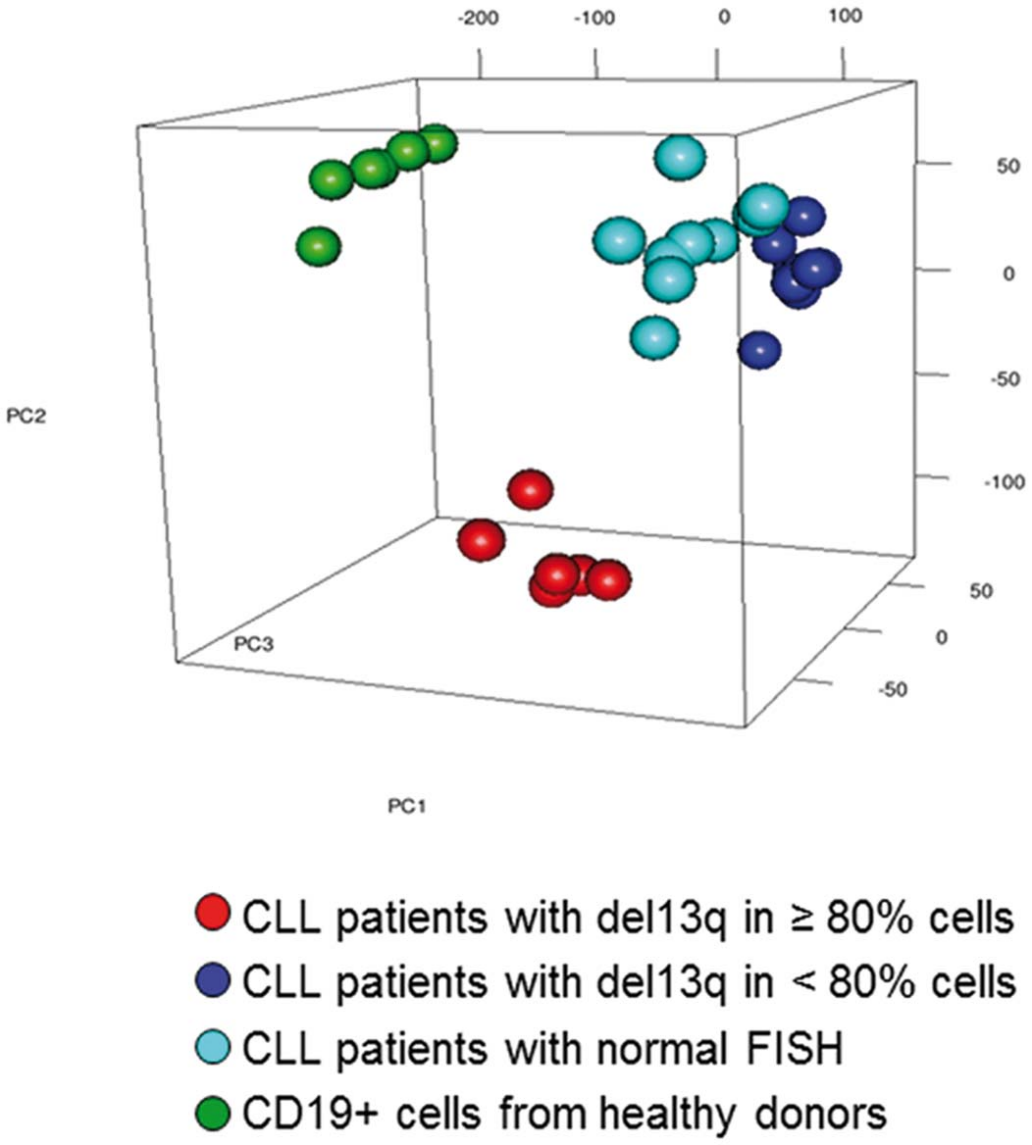
CLL patients with a high number of 13q- cells can be differenciated based on their expression profile. Principal component analysis (PCA) plot of CD19+cells from healthy controls (green), CLL with normal FISH (sky blue), 13q-H CLL (red) and 13q-L CLL (dark blue) was carried out using the 28,806 remaining genes after filtering the normalized gene expression matrices to remove the least variable genes (25%). Each sphere represents a single GEP. The result of the PCA shows a cumulative variance of 48.3%, 60.9% and 68.3% corresponding to one, two or three of the initial components, respectively. The expression pattern of CD19+cells from CLL patients is notably different from the gene expression profile of CD19+cells from healthy donors. Of note, the PCA analysis shows that 13q-H CLL patients have a distinctive gene expression profile. By contrast, the gene expression of B lymphocytes from 13q-L CLL and nCLL was similar.

Overall, the expression pattern of B lymphocytes from 13q-H and 13q-L CLL patients and nCLLs was notably different from the gene expression profile of B lymphocytes from healthy donors, as expected (Figure 5). PCA revealed a cumulative variance between groups of 48.3%, 60.9% and 68.3% corresponding to one, two and three of the initial components, respectively. Since the first three principal components explained a considerable proportion of the overall variance (68%), the 3D representation was able to show the main similarities and differences between categories. Notably, the 13q-H samples were largely separated from the others. Thus, 13q-H patients had a distinctive GEP that was different not only from healthy donors but also from all other CLLs, including 13q-L patients. By contrast, the gene expression of B lymphocytes from 13q-L CLL and nCLL was similar (Figure 5). SAM analysis revealed differences in the expression of 15 332 and 16 754 genes between CD19+cells from 13q-L or nCLL compared with B lymphocytes from healthy donors, respectively, while both subgroups (13q-L and nCLL patients) shared the deregulation of 13 749 genes (data not shown). Moreover, the analysis failed to demonstrate differences between nCLL and 13q-L patients, while 131 genes were differentially expressed in comparison with 13q-H (data not shown).

Thus, both qualitatively (PCA) and quantitative (SAM) analysis showed that the gene expression profile of 13q- CLLs is different depending on the percentage of cells displaying this aberration.

### IgVH Mutational Status and Mono/biallelic 13q14 Deletion in 13q-patients

Given that the prognostic significance of IgVH mutations is independent from that of cytogenetic abnormalities, we also analyzed the IgVH mutational status in the 13q- subgroups. There was no significant difference between both 13q- subgroups (*P* = 0.664).

Regarding the distribution of biallelic 13q14 deletion in both 13q- subgroups, no correlation between the presence of biallelic 13q14 deletion and the percentage of 13q- cells was observed. Thus in the group of patients with 3 of the 32 cases (9%) had a biallelic loss of 13q, while in the group of 13q-L 5 of 38 patients (13%) showed a biallelic loss on 13q. (*P = *N.S.).

## Discussion

13q deletion (13q-) is the most common cytogenetic aberration in CLL and it is usually associated with the most favourable prognosis as a sole abnormality [5]. However, recent studies have shown that CLL patients carrying higher percentages of 13q- cells have more aggressive clinical courses [11]–[13]. By combining gene expression profile and miRNA analysis, we have shown that 13q- patients are also a biologically heterogeneous group, in which a higher number of 13q- cells (13q-H) could involve the deregulation of relevant cellular pathways. Thus, several pathways are involved in 13q-H patients (Table 2 and Table S3), BCR signaling, NFkB signaling and antiapoptotic pathways being of special interest in CLL. Deregulation of several miRNAs (Table 1) was also observed. The influence of other factors with prognostic relevance in CLL, such as IGVH mutational status, was discarded.

The BCR is an essential signal transduction pathway for the survival and proliferation of mature B lymphocytes. In the present study, monoclonal B-cells in 13q-H CLL patients exhibit a molecular signature characterized by the overexpression of genes mainly involved in BCR signaling (Figure 1). There is now strong evidence that signaling via the B cell receptor plays a major role in the development of CLL, and it could be related to the different clinical outcomes of CLL [26]. Thus, the BCR pathway is activated in poor prognosis CLL patients (IGHV unmutated), and the overexpression of several molecules involved in this pathway has been reported in advanced stages of the disease [27], [28]. In addition, SYK expression is enhanced in CLL relative to healthy B cells and also in unmutated compared with mutated CLL, possibly reflecting the increased BCR signaling in these patients [29]. In our study 13q-H CLL also overexpressed *SYK* (Figure 1), providing new evidence of the involvement of the BCR pathway in this group of CLLs. In addition, this group of patients also showed upregulation of *CD79b.* Chronic active BCR signaling due to point mutations in *CD79b* has recently been identified as a key pathogenic mechanism in aggressive B-cell lymphoma, and results in constitutive nuclear factor-kB (NF-kB) activation [30]. Interestingly, CLL patients with deletions on 17p or 11q or those with losses in 13q in a high percentage of cells had an increased expression of a cluster of genes comprising several PKCs, such as *PRKCB1* and *PRKCZ.* Previous studies have shown an overexpression of *PKC* in human CLLs, which is part of a poor-prognosis gene cluster in CLL linked to the transmission of BCR signals such as calcineurin-NFAT and NF-kB, which our analysis also revealed to be deregulated (Table 2) [31], [32]. Furthermore, the overexpression of calcium metabolism-related genes as well as several MAPK in 13q-H patients was also observed in the present study, which would be consistent with these previous studies (Table 2).

One of the hallmarks of this clinically heterogeneous disease is defective apoptosis, which is considered to contribute not only to cell accumulation but also to disease progression and resistance to therapy [26]. In this study we report the overexpression of genes involved in promoting cell survival and antiapoptotic pathways, as well as the downregulation of several proapoptotic genes in 13q-H CLL patients (Table 2 and Table S3). We confirm the overexpression of *LEF-1* in CLL B cells compared with B cells from healthy donors (data not shown), as previously reported [33], but we also observed upregulation of *LEF-1* and other genes involved in the Wnt signaling pathway in 17p-, 11q- and 13q-H patients in comparison with 13q-L cases. Wnt pathway gene expression is widely known to be deregulated in CLL [34], [35]. Alterations of RAS signaling are associated with potent oncogenic effects, which keep the cell in a proliferative state and block apoptosis, thereby paving the way for cancer formation. Overexpression of *RRAS* and other molecules involved in this signaling cascade, such as SOS1, RHOC and several MAP kinases, was also observed. In addition, apoptosis was also deregulated in 13q-H patients by the involvement of both mitochondrial (*BCL2* and several caspases) and extrinsic (*FAS*) pathways. Interestingly, the apoptotic signature of 13q-H patients showed a similar pattern of deregulation to that of high-risk cytogenetic groups (Figure 4B), since they both featured the alteration of several genes involved in the classic apoptotic pathway (mitochondrial). Sustained BCR signaling has also been reported to have an antiapoptotic effect [36]. Thus, in 13q-H CLL patients, our study shows an imbalance between the proliferative and apoptotic signals, which could explain the higher level of lymphocytosis and the poor outcome previously described in these patients [11].

An aberrant cellular miRNA expression profile in CLL cells has already been described and the changes correlate well with prognostic factors, including ZAP-70 expression status and *IgVH* mutations in CLL patients [37]. A recent study evaluating microRNAs as a signature for CLL patients with specific chromosomal abnormalities found nine miRNAs whose expression values were correlated with a specific karyotype [38]. In our study we found that several miRNAs were deregulated in 13q-H patients (Table 1), some of which had been previously reported in CLL (Table 3). Several important miRNAs, such as miR223, miR-29a and miR-181, were downregulated in 13q-H and high-risk cytogenetic subgroups, which could be related to the worse outcome in these groups of patients [39], [40]. By contrast, overexpression of miR-155 was observed, which could be related to enhanced BCR-activation, as previously reported [41]. The pathogenic role of deletion 13q in CLL has been related to the lack of B-cell proliferation control allegedly determined by the deletion of the *DLEU2/MIR15A/MIR16-1* locus [42]. Interestingly, miR-15a was downregulated in 13q-H CLL patients and it has been reported to induce apoptosis through the negative regulation of *BCL2,* overexpressed in the 13q-H group of patients. It should be noted that a third of deregulated genes in 13q-H compared with 13q-L were putative targets of miRNAs also altered in this analysis, supporting the presence of a specific relationship between miRNA and gene expression in 13q-H CLL patients. Most of these genes were related to TGF or BCR signaling and confirmed these pathways to be those most commonly affected by miRNA deregulation in 13q-H patients. Among the putative target mRNAs we found many genes, such as *TCL1A, BCL2, LEF1* [33], [43], [44], to be closely involved in CLLs (Table 3). These results suggest that miRNAs have a key role in the reported heterogeneity of 13q- patients. Surprisingly, our results suggest that some of the biological characteristics of 13q-H CLL patients are similar to those of high-risk cytogenetic subgroups, since they share the deregulation of several key signaling pathways (Figure 4B; Table S6). However, 13q-L patients had similar gene expression to that of CLL with normal FISH (Figure 5).

Therefore, this study provides new evidence regarding the heterogeneity of 13q deletion in CLL patients, showing that apoptosis, BCR and NF-kB signaling as well as miRNA regulation are the most significant affected pathways in 13q-H CLL patients. The identification of the mechanisms responsible for the clinical heterogeneity of CLL, including the mutations recently described [45], [46] and the critical signaling pathways affected can lead to a better understanding of the molecular pathogenesis of the disease.

## Supporting Information

Figure S1 **Summary of the miRNA analysis performed in the study.** The chart explains the steps involved in the identification and validation of the miRNAs and their deregulated targets in 13q- CLL patients.
(TIF)

Figure S2 **Box plot of the expression levels of three genes with significant differences between 13q-H and 13q-L patients, assessed by semi-quantitative PCR.** Box plots show the values for *GAS7*, *E2F1* and *FCRL2* relative expression [represented as arbitrary units (a.u.)], showing a significant difference in the level of expression between 13q-H and 13q-L CLL patients. The thick line inside the box plot indicates median expression levels, the limits of the box represent the 25th and 75th percentiles, and the whiskers show the maximum and minimum values. Outliers (extreme values falling outside the main distribution) are represented by open circles. Statistical significance was determined using the Mann-Whitney U test (*P*<0.05).
(TIF)

Figure S3 **BCR signaling pathway identified as the top canonical pathway altered in CLL patients with higher percentages of 13q- losses according to the Ingenuity Pathway Analysis knowledge base.** Genes significantly differentially expressed between CLL with 80% or more of cells with loss of 13q (13q-H) and CLL with losses in 13q in fewer than 80% of cells (13q-L) were mapped to the pathway and colored in red if the expression levels were higher, or in green if they were lower in 13q-H than in 13q-L cases. Significant positions of the pathway are occupied by genes deregulated in our analysis, indicating that this pathway is affected in 13q-H patients. CLL patients with 17p and 11q deletions showed similar deregulation in this pathway.
(TIF)

Figure S4 **Overlap of differentially expressed genes as analyzed by SAM.** Venn diagram illustrating the number of significantly affected genes in common and distinct for the contrasts (1) and (2). 13q-H and 17p−/11q- shared the deregulation of 46% of genes (n = 1325) relative to 13q-L.
(TIF)

Table S1 Clinical and biological features of CLL patients included in the study.
(XLS)

Table S2 Sequences of primers used for SYBR Green detection.
(XLS)

Table S3 Enriched functional annotations terms associated with the 3450 differentially expressed genes in 13q-H CLL patients. Genes were clustered into functional categories using the DAVID Bioinformatics Database Gene Functional Classification Tool (NIAID/NIH). The P-value is provided by DAVID bioinformatics resources.
(XLS)

Table S4 miRNAs and their predicted targets (n = 1027) that are significantly deregulated in 13q-H CLL patients. By applying an integrated miRNA-mRNA analysis, mRNA targets were identified for the list of miRNAs deregulated in 13q-H CLL patients. The P-value for each predicted target gene refers to the contrast between 13q-H and 13q-L CLL patients.
(XLS)

Table S5 Functional analysis of the potential target genes of the deregulated miRNAs in CLL patients with a high number of 13q- cells (13q-H). The 432 mRNA target genes that showed an inverse relationship with miRNA expression level were input into Ingenuity (Ingenuity Systems, Inc.) and core analysis was then performed to retrieve the target genes’ association with biological functions of relevance in CLL.
(XLS)

Table S6 Most significant differentially expressed genes in patients with 80% or more cells showing 13q- (13q-H) and 17p/11q deletion compared with 13q-L patients. (Upper: overexpressed; Lower: underexpressed in 13q-H, 17p- and 11q- patients with respect to the 13q-L CLL patients).
(XLS)
