# Supplementary figures and images for: Molecular Characterization of Chronic Lymphocytic Leukemia Patients with a High Number of Losses in 13q14
Source: PLoS One. 2012 Nov 13;7(11):e48485. doi: 10.1371/journal.pone.0048485 (PMC3496725; doi:10.1371/journal.pone.0048485)

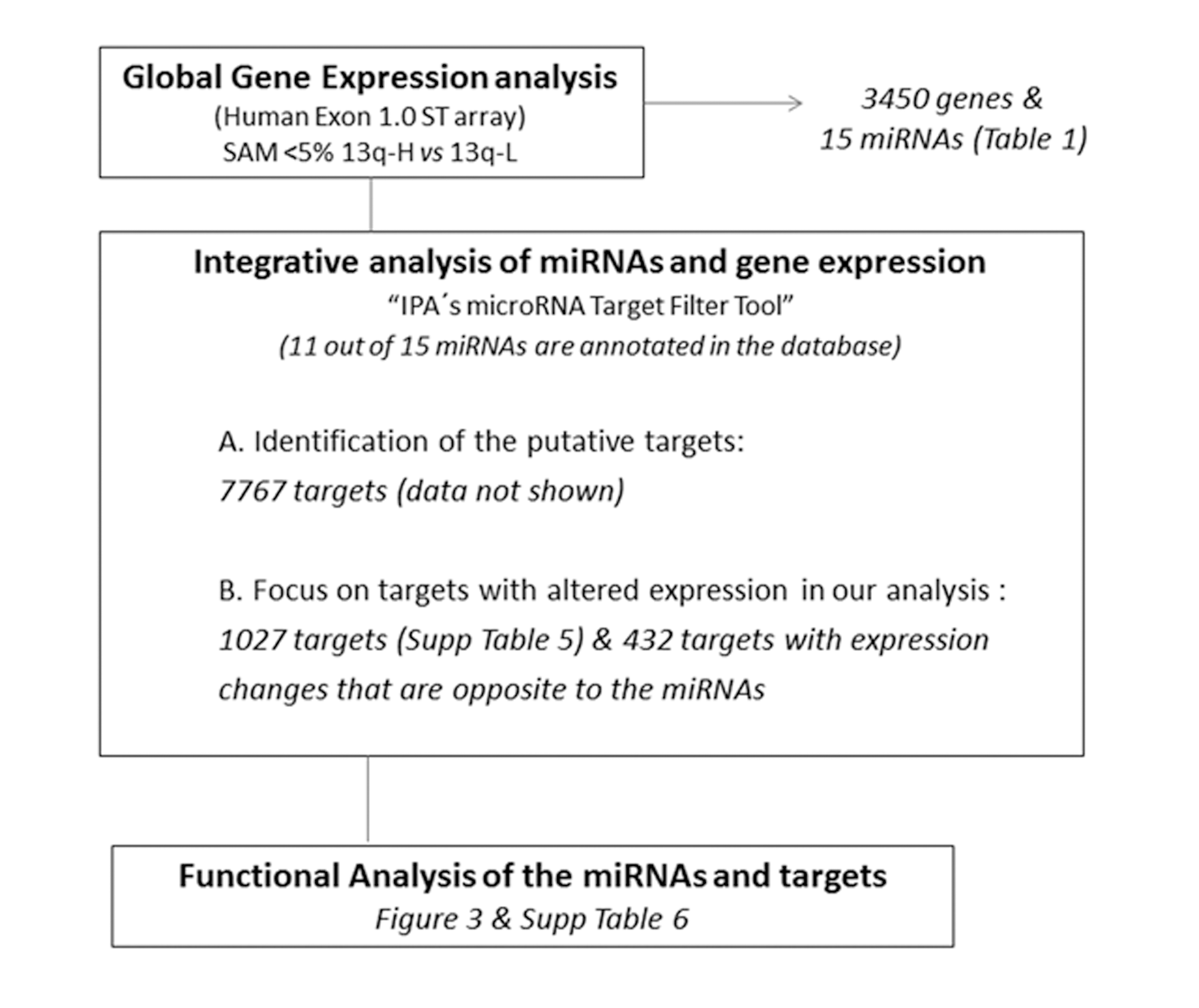

Supplement: Figure S1 — Summary of the miRNA analysis performed in the study. The chart explains the steps involved in the identification and validation of the miRNAs and their deregulated targets in 13q- CLL patients. (TIF) [file pone.0048485.s001.tif]

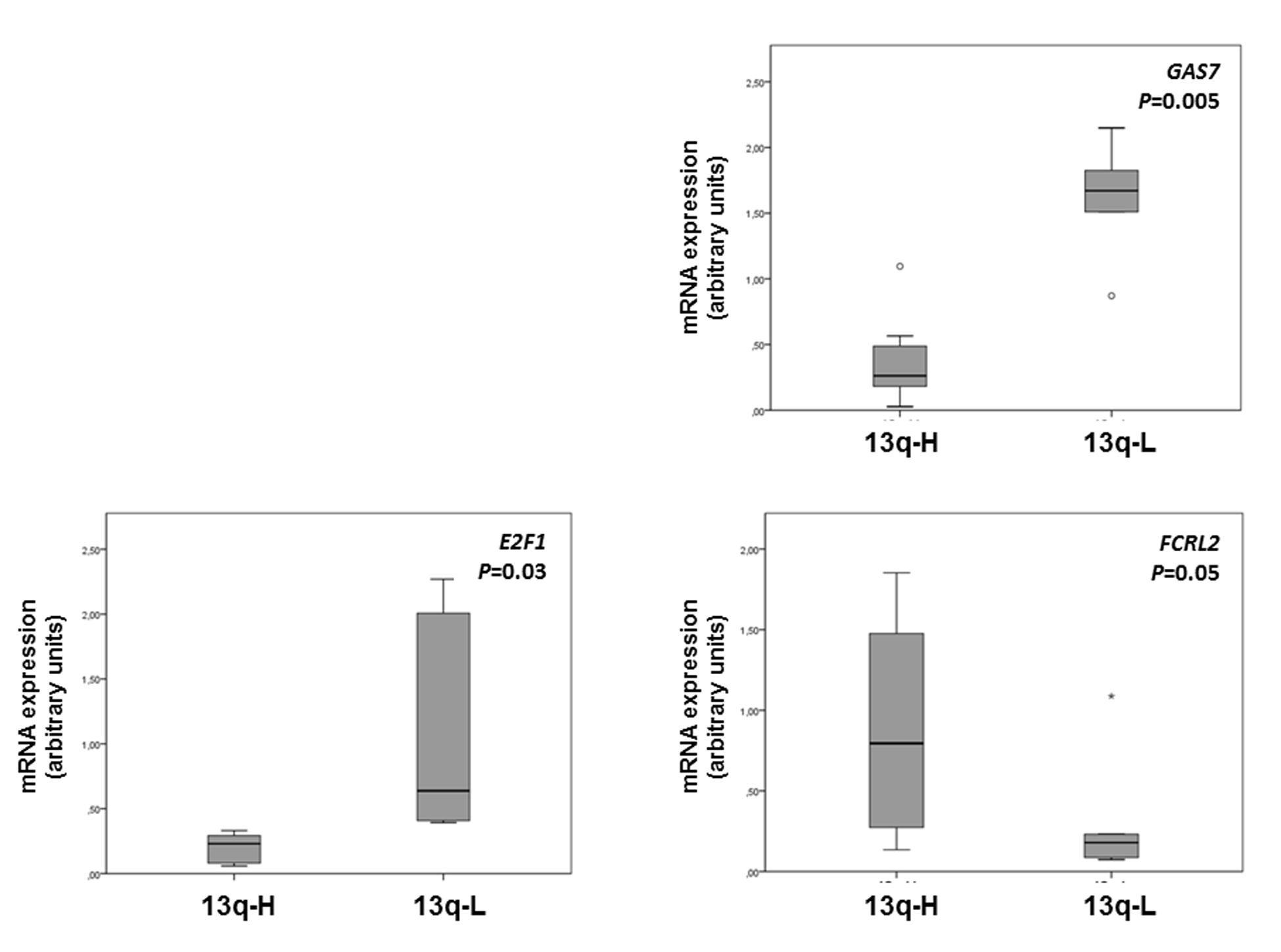

Supplement: Figure S2 — Box plot of the expression levels of three genes with significant differences between 13q-H and 13q-L patients, assessed by semi-quantitative PCR. Box plots show the values for GAS7, E2F1 and FCRL2 relative expression [represented as arbitrary units (a.u.)], showing a significant difference in the level of expression between 13q-H and 13q-L CLL patients. The thick line inside the box plot indicates median expression levels, the limits of the box represent the 25th and 75th percentiles, and the whiskers show the maximum and minimum values. Outliers (extreme values falling outside the main distribution) are represented by open circles. Statistical significance was determined using the Mann-Whitney U test (P<0.05). (TIF) [file pone.0048485.s002.tif]

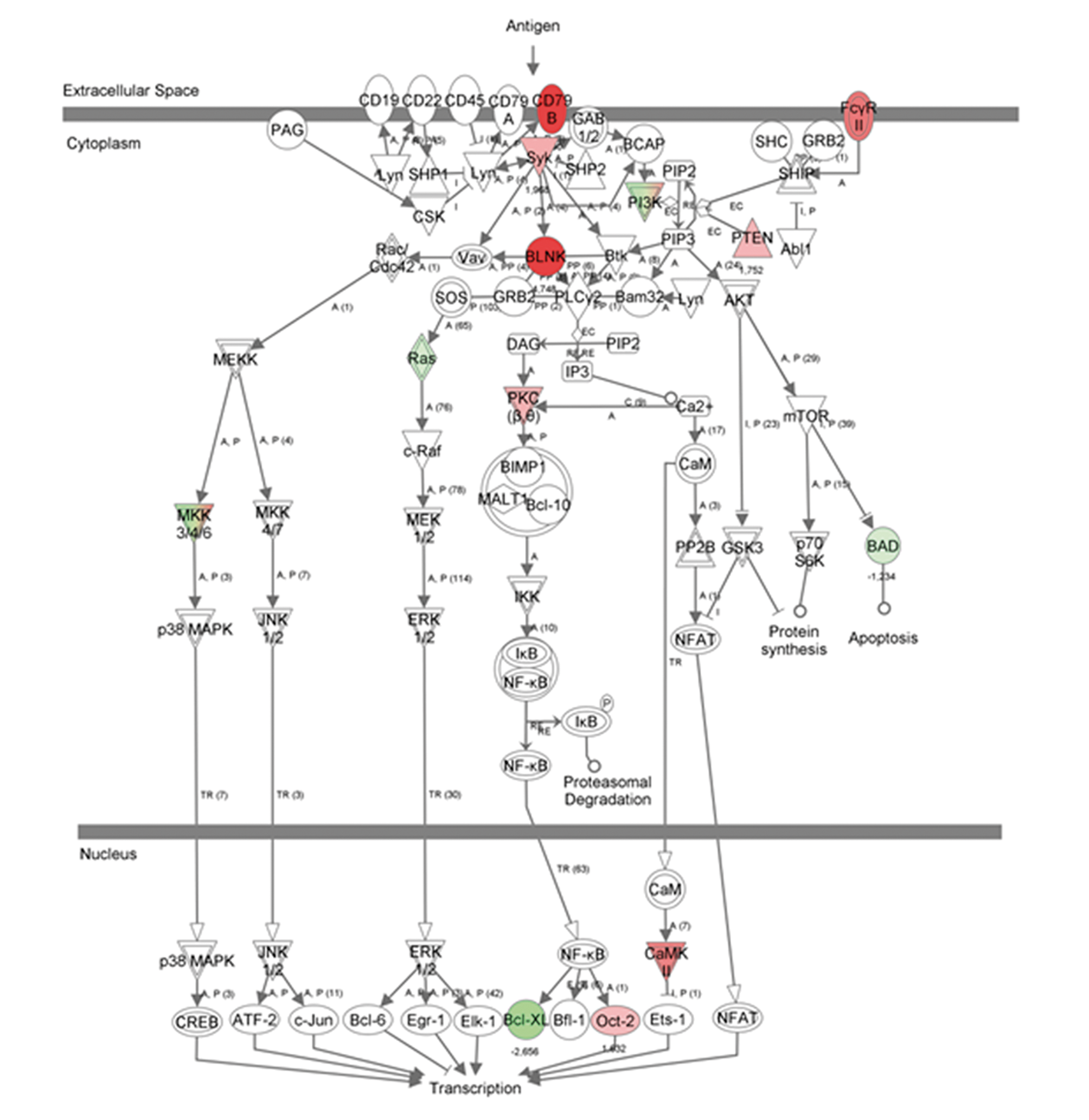

Supplement: Figure S3 — BCR signaling pathway identified as the top canonical pathway altered in CLL patients with higher percentages of 13q- losses according to the Ingenuity Pathway Analysis knowledge base. Genes significantly differentially expressed between CLL with 80% or more of cells with loss of 13q (13q-H) and CLL with losses in 13q in fewer than 80% of cells (13q-L) were mapped to the pathway and colored in red if the expression levels were higher, or in green if they were lower in 13q-H than in 13q-L cases. Significant positions of the pathway are occupied by genes deregulated in our analysis, indicating that this pathway is affected in 13q-H patients. CLL patients with 17p and 11q deletions showed similar deregulation in this pathway. (TIF) [file pone.0048485.s003.tif]

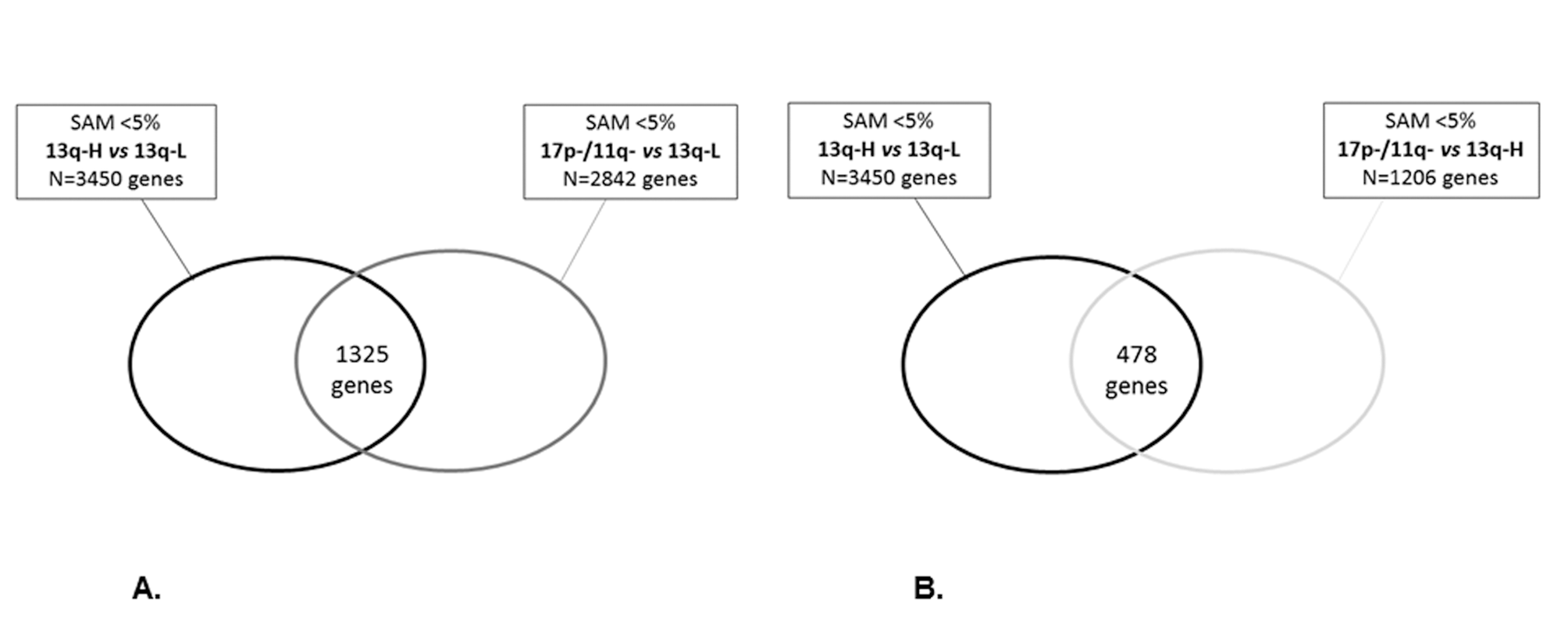

Supplement: Figure S4 — Overlap of differentially expressed genes as analyzed by SAM. Venn diagram illustrating the number of significantly affected genes in common and distinct for the contrasts (1) and (2). 13q-H and 17p−/11q- shared the deregulation of 46% of genes (n = 1325) relative to 13q-L. (TIF) [file pone.0048485.s004.tif]
